# Natural history of familial cerebral cavernous malformation syndrome in children: a multicenter cohort study

**DOI:** 10.1007/s00234-022-03056-y

**Published:** 2022-10-06

**Authors:** Ana Filipa Geraldo, Cesar Augusto P. F. Alves, Aysha Luis, Domenico Tortora, Joana Guimarães, Daisy Abreu, Sofia Reimão, Marco Pavanello, Patrizia de Marco, Marcello Scala, Valeria Capra, Rui Vaz, Andrea Rossi, Erin Simon Schwartz, Kshitij Mankad, Mariasavina Severino

**Affiliations:** 1 Diagnostic Neuroradiology Unit, Department of Radiology, Centro Hospitalar Vila Nova de Gaia/Espinho (CHVNG/E), Vila Nova de Gaia, Portugal; 2 Clínica Universitária de Imagiologia, Faculty of Medicine of the University of Lisbon, Lisbon, Portugal; 3 Department of Radiology, Children’s Hospital of Philadelphia, Philadelphia, PA USA; 4 Department of Radiology, Great Ormond Street Hospital for Children, NHS Foundation Trust, London, UK; 5 Department of Radiology, King’s College London, London, UK; 6 Neuroradiology Unit, IRCCS Istituto Giannina Gaslini, Genova, Italia; 7 Department of Neurology, Centro Hospitalar Universitário de São João, Porto, Portugal; 8 Department of Clinical Neurosciences and Mental Health, Faculty of Medicine of the University of Porto, Porto, Portugal; 9 Instituto de Medicina Molecular João Lobo Antunes, Lisbon, Portugal; 10 Neurological Imaging Department, Hospital de Santa Maria, Lisbon, Portugal; 11 Neurosurgery Unit, IRCCS Istituto Giannina Gaslini, Genoa, Italy; 12 Medical Genetics Unit, IRCCS Istituto Giannina Gaslini, Genoa, Italy; 13 Department of Neurosciences, Rehabilitation, Ophthalmology, Genetics, Maternal and Child Health, Università Degli Studi Di Genova, Genoa, Italy; 14 Pediatric Neurology and Muscular Diseases Unit, IRCCS Istituto Giannina Gaslini, Genoa, Italy; 15 Neurosurgical Department, Centro Hospitalar Universitário de São João, Porto, Portugal; 16 Department of Health Sciences (DISSAL), University of Genoa, Genoa, Italy

**Keywords:** Cavernous malformation, Familial cavernous malformation syndrome, Magnetic resonance imaging, Brain imaging

## Abstract

**Purpose:**

There is limited data concerning neuroimaging findings and longitudinal evaluation of familial cerebral cavernous malformations (FCCM) in children. Our aim was to study the natural history of pediatric FCCM, with an emphasis on symptomatic hemorrhagic events and associated clinical and imaging risk factors.

**Methods:**

We retrospectively reviewed all children diagnosed with FCCM in four tertiary pediatric hospitals between January 2010 and March 2022. Subjects with first available brain MRI and $\ge$ 3 months of clinical follow-up were included. Neuroimaging studies were reviewed, and clinical data collected. Annual symptomatic hemorrhage risk rates and cumulative risks were calculated using survival analysis and predictors of symptomatic hemorrhagic identified using regression analysis.

**Results:**

Forty-one children (53.7% males) were included, of whom 15 (36.3%) presenting with symptomatic hemorrhage. Seven symptomatic hemorrhages occurred during 140.5 person-years of follow-up, yielding a 5-year annual hemorrhage rate of 5.0% per person-year. The 1-, 2-, and 5-year cumulative risks of symptomatic hemorrhage were 7.3%, 14.6%, and 17.1%, respectively. The latter was higher in children with prior symptomatic hemorrhage (33.3%), *CCM2* genotype (33.3%), and positive family history (20.7%). Number of brainstem (adjusted hazard ratio [HR] = 1.37, *P* = 0.005) and posterior fossa (adjusted HR = 1.64, *P* = 0.004) CCM at first brain MRI were significant independent predictors of prospective symptomatic hemorrhage.

**Conclusion:**

The 5-year annual and cumulative symptomatic hemorrhagic risk in our pediatric FCCM cohort equals the overall risk described in children and adults with all types of CCM. Imaging features at first brain MRI may help to predict potential symptomatic hemorrhage at 5-year follow-up.

**Supplementary Information:**

The online version contains supplementary material available at 10.1007/s00234-022-03056-y.

## Introduction

Cerebral cavernous malformations (CCM) are low-flow venous-capillary parenchymal brain lesions composed of enlarged, multilobulated, and leaky blood-filled sinusoidal spaces devoid of mature vascular walls and without intervening brain parenchyma [1]. Clinical manifestations of CCM may occur at any age, and include epileptic seizures, impaired consciousness, focal neurologic deficits, and headaches. In addition, asymptomatic presentation in the context of imaging screening or incidental detection of CCM may also occur. Overall, up to 25% of all CCM manifest in childhood [1, 2], and these vascular lesions represent one of the major causes of non-traumatic acute intracranial hemorrhage in this age group [3].

CCM are usually classified as familial (FCCM) or sporadic [1, 2]. Although histopathologically indistinguishable, these conditions usually have distinct genetic signatures. Indeed, FCCM (accounting for up to 20% of all CCM) is an autosomal dominant inherited disease with incomplete penetrance caused by inherited heterozygous germline loss-of-function pathogenic variants in *CCM1-3 genes* [4]. Sporadic CCM are instead mainly due to activating somatic mutations in genes involved in the PI3K-AKT-mTOR pathway, especially *PIK3CA* and *MAP3K3* [5–7]. Also, unlike sporadic CCM, familial forms often have a positive family history and the disease manifestations tend to present at an earlier age, usually in the form of multiple, scattered CCM [1, 8] as well as other systemic CM [9, 10].

Although the natural history of CCM in the general population has been relatively well studied [8, 11, 12], there are limited knowledge and conflicting results concerning the longitudinal evolution of pediatric FCCM. Indeed, most papers focusing on the natural history of CCM in familial cases include mixed-adult and pediatric populations and were published before 2008 [13–17], the year when a consensus statement regarding definitions and reporting standards of CCM-related hemorrhage was issued [18]. In addition, prior to 2008, gradient recovery echo-related sequences including T2* and SWI (considered the gold standard for in vivo assessment of CCM) were uncommonly used in clinical practice and frequently excluded from the analysis [13–17]. Moreover, the few available studies focusing on younger subjects with CCM are often based on cohorts including both children and young adults and/or all subtypes of CCM [19–23]. Indeed, familial cases only tend to account for a small proportion of the complete pediatric patients, with limited data available concerning their clinical features and imaging findings and underlying genotype [19–23]. All these limitations introduce sample heterogeneity and raise concerns about the appropriateness of extrapolating results to the pediatric FCCM population.

Characterization of pediatric FCCM is relevant for the prognostic assessment of affected children and the development of a more personalized risk–benefit assessment in this population in terms of treatment options, especially regarding potential new disease-modifying pharmacologic agents currently under investigation [24–26].

Our aim was to study the natural history of pediatric FCCM, with an emphasis on symptomatic hemorrhagic events and associated clinical, genetic, and imaging risk factors.

## Material and methods

### Population

We performed a multicenter retrospective cohort study involving four tertiary pediatric institutions including all consecutive subjects with a diagnosis of FCCM during childhood (≤ 18 years) evaluated at least once in the participating hospitals between January 2010 and September 2021, and with an initial brain MRI available for review at least including axial T1WI, T2WI, and GRE-type sequences (T2* and/or SWI). The diagnosis of FCCM was based on (1) the presence of $\ge$ 1 CCM associated with either a positive family history (defined as $\ge$ 1 known first or second-degree relative with a proven CM) and/or (2) a confirmed pathogenic variant in the *CCM1-3* genes in the affected patient or in a first-degree relative [27]. Exclusion criteria included (1) history of prior radiation, severe head trauma requiring hospitalization, extracorporeal membrane oxygenation support, intensive care unit stay, and/or anticoagulation therapy and (2) poor-quality or incomplete brain MR.

### Genetic analysis

Details on genetic analysis are presented in the Online Supplemental Data.

### MR technique and image analysis

Images were acquired with a 1.5 or a 3.0 Tesla MR scanner, using local protocols, in some subjects including administration of gadolinium-based contrast agents. For each subject, lesion counts were performed on the first brain MRI based on hemosiderin-sensitive sequences (T2* and/or SWI) and the anatomical location. They were assessed for Zabramski type as modified by Nikoubashman [13, 21] and size (corresponding to the largest diameter measured on axial T2WI) [21]. Lesions larger than 40 mm were classified as giant CCM, according to previous definitions [28]. Longitudinal studies were assessed whenever available and correlated with clinical indications, focusing on the appearance of new CCM (de novo lesions) and evidence of symptomatic hemorrhage, when present. CCM were labeled as de novo lesions if their new appearance could be shown on comparable or technically inferior consecutive studies while acute symptomatic hemorrhagic CCM (CASH) at either presentation and/or follow-up were identified according to previously published consensus guidelines [18]. These lesions were further evaluated in relation to their general morphology (uni/multilocular), as well as the presence of hemosiderin ring, perilesional vasogenic edema, T1 hyperintense perilesional sign [29], fluid–fluid levels, and/or accompanying developmental venous anomalies (DVA). In cases with clinical symptoms and more than one CCM with signs of recent hemorrhage, the lesion located in the anatomical region corresponding to the neurological manifestations was considered. If the clinico-radiological correlation was uncertain or dubious, the largest hemorrhagic lesion was considered for evaluation.

At each center, images were first analyzed by one rater, blinded to the clinical and genetic information. One of the raters evaluated two sites, corresponding to a total of 3 readers (with 1, 7, and 7 years of experience in pediatric neuroradiology, respectively). Anonymized brain MR studies of all potentially CASH and of other CCM with questionable evaluation were decided by consensus.

### Clinical data

Data on demographics, age at clinical presentation leading to the FCCM diagnosis and mode of presentation, ethnicity, family history (including the number of affected family elements and degree of kinship), genotype, presence of other systemic CM (including cutaneous, retinal, or within solid organs), or any combined systemic feature (including Greig cephalopolysyndactyly in the setting of a 7p deletion syndrome [30]) were obtained from medical records. The mode of the presentation was classified according to previously published reporting standards of CCM [18]. More specifically, symptomatic CCM hemorrhage was defined by the presence of acute or subacute onset symptoms (any of headache, epileptic seizure, impaired consciousness, or new/worsened focal neurological deficit referable to the anatomic location of the CM) accompanied by radiological evidence of recent extra- or intralesional hemorrhage. Other types of presentation included non-hemorrhagic epilepsy, non-hemorrhagic focal neurological deficit, non-hemorrhagic unspecific headaches, or asymptomatic forms (such as incidental finding or detection in the context of imaging screening due to family history of CCM). Follow-up data were obtained through routine visits in specialized outpatient clinics and by presentation in the emergency departments, including symptomatic events attributable to CCM according to current guidelines [18] after confirmation by multidisciplinary assessment. In addition, any neurosurgical interventions and final neurological outcomes (graded as normal, mild, moderate, or severe impairment) were also recorded.

### Statistical analysis

Quantitative data were presented as median and interquartile range, and categorical data as frequencies and percentages. Fisher’s exact test or Pearson’s *χ*^2^ test and Student’s *t*-test or the Mann–Whitney test were used to compare categorical and continuous variables, respectively.

The prospective annual risk of hemorrhage was calculated as the number of hemorrhages during considered follow-up divided by person-years of follow-up during that time. Cumulative rates of symptomatic hemorrhage for the whole sample and stratified by baseline variables were calculated as the ratio between the number of symptomatic hemorrhagic events during follow-up and the number of patients initially at risk. Cumulative rates of symptomatic hemorrhage were also illustrated using the Kaplan–Meier method, and the curves were compared by the log-rank test. Survival analysis was applied to estimate the 1-year risk, 2-year risk, and 5-year risk of symptomatic hemorrhage with corresponding 95% confidence intervals (95CI). Univariable and multivariable Cox regression survival analyses were used to identify risk factors of longitudinal symptomatic hemorrhage during the follow-up period.

Data were excluded if subjects experienced bleeding or were lost to follow-up. Surgical removal of a lesion did not lead to exclusion if the patient had additional CCM(s) amenable to follow-up.

Statistical analyses were performed by using Stata, v14.0 (StataCorp, College Station, Texas). The significance level was set at *P* = 0.05 (2-sided).

### Data availability statement

Any data not published within the article will be shared, in anonymized form, by request from any qualified investigator.

## Results

### Clinical and genetic data

Fifty-three children with FCCM from 49 families were identified, from which 12 patients (22.6%) were excluded due to non-available first brain MRI. Forty-one children from 38 unrelated families fulfilled the eligibility criteria and were therefore included. Clinical and genetic data are summarized in Table 1. Twenty-two individuals (53.7%) were male and 30 (73.2%) Caucasian. There was a family history in 29 (70.7%) children, with multiple kindred involved in 18 (63.1%). A genetic diagnosis in the affected individual or in a known first-degree relative was available in 30 (73.2%) children, with *CCM1*, *CCM2*, and *CCM3* loss-of-function variants identified in 17 (56.7%), 6 (20.0%), and 7 (23.3%), respectively*.* Out of the six subjects with *CCM2* abnormalities, five (83.3%) had a 7p deletion, including two cases with features of Greig cephalopolysyndactyly.

**Table 1:** Demographic, genetic, clinical, and spine imaging data

	*N* = 41
Male, *n* (%)	22 (53.7)
Age at initial clinical presentation in years, median (IQR)	7.7 (3.47–12.67) Range: 0.4–17.3
Genotype, *n* (%)	
C*CM1*	17 (41.5)
CCM2^a^	6 (14.6)
C*CM3*	7 (17.1)
* CCM1-3* testing negative/not performed/pending	11 (26.8)
Positive family history, *n* (%)	29 (70.7)
No. of affected family members	
* n* = 1	11 (37.9)
* n* = 2	11 (37.9)
$\ge$ 3	7 (24.1)
Ethnic origin, *n* (%)	
Caucasian	30 (73.2)
African	5 (12.2)
Asian	2 (4.9)
Hispanic	1 (2.4)
Other	3 (7.2)
Presentation mode, *n* (%)	
Symptomatic hemorrhage of the CNS^b^	15 (36.6)
Non-hemorrhagic seizures	10 (24.4)
Incidental diagnosis	6 (14.6)
Imaging screening	4 (17.1)
Headache	3 (7.2)
$\ge$ 1 extra-CNS CM, *n* (%)	2 (4.9)
$\ge$ 1 spinal cord CM, *n* (%)	3 (13.6) ^c^
$\ge$ 1 brain surgery, *n* (%)	19 (46.3)
Number of neurosurgical procedures	
* n* = 1	17 (89.5)
* n* = 2	2 (10.5)
Neurological assessment at last clinical FU, *n* (%)	
Normal	29 (70.7)
Mild impairment	3 (73)
Moderate impairment	6 (14.6)
Severe impairment	2 (4.9)
Death	1 (2.4)
Seizures at last clinical FU, *n* (%)	12 (29.3)
Medically controlled	10 (83.3)
Medically refractory	2 (16.7)

Legend: *CM*, cavernous malformation; *CNS*, central nervous system; *FU*, follow-up; *IQR*, interquartile range; *SD*, standard deviation

^a^Includes 4 cases with a 7p deletion; ^b^1 case due to a SCCM-related hemorrhage, ^c^22/41 subjects (53.7%) underwent at least one whole-spine MR

The median age at clinical presentation leading to subsequent FCCM diagnosis was 7.7 years (IQR = 9.2; range: 0.4–17.3). Fifteen (36.6%) individuals suffered a symptomatic CCM-related hemorrhage of the central nervous system at presentation, intracranial in 14 (93.3%) cases, and involving the spinal cord in one (6.7%).

The median observational period was 54.3 months (IQR: 65.0; range: 4.0–205.4). All subjects except one (*n* = 40, 97.6%) had more than 6 months of clinical follow-up. During extended, retrospective longitudinal evaluation, 19 (46.3%) subjects underwent at least one CCM-related brain surgery (total of 21 CCM-excisional procedures), with complete removal in 17 (81.0%) and subtotal removal in 4 (19.1%) lesions. Eighteen (94.7%) surgeries were within the first 5 years after diagnosis. No spinal surgeries were performed. The mean age at first surgery was 9.1 (SD = 5.2; range: 0.9–17.2). Overall, 11 out of 19 (52.4%) procedures were performed due to a CCM-related symptomatic hemorrhage. Other surgical indications included progressive CCM growth with or without asymptomatic hemorrhage (*n* = 3, 14.3%), medically refractory seizures related to a surgically accessible lesion (*n* = 5, 23.8%), and a giant CCM (*n* = 2, 9.5%). Children undergoing more than 1 surgical CCM removal had a significantly higher rate of symptomatic hemorrhage at presentation (*P* = 0.047) and a significantly lower number of CCM on the first brain MRI (*P* = 0.0204) when compared to those receiving conservative treatment. Otherwise, there were no statistically significant differences between groups regarding other studied demographic, clinical, and brain imaging variables (Supplemental Table 1).

At their last clinical visit, 29 (70.7%) children remained neurologically intact, while 11 (26.8%) demonstrated some type of neurological impairment. One (2.4%) child died during the follow-up due to sudden-unexpected death in the context of severe medically refractory epilepsy. Within the subgroup with mild to severe neurological impairment, *n* = 5 (45.5%) subjects had $\ge$ 1 previous symptomatic hemorrhagic event, *n* = 1 (9%) presented refractory epileptic encephalopathy, and *n* = 2 (18.2%) showed Greig cephalopolysyndactyly–related developmental delay.

### Brain MR and CCM

Details on neuroimaging studies and protocols are presented in the Online Supplemental Data. At first brain MRI, a total of *n* = 587 CCM were identified on T2* and/or SWI sequences and their neuroimaging features are reported in Table 2. All children except one (97.6%) demonstrated multiple CCM (median number per child = 10.0; IQR = 12; range: 1–80) at diagnosis. CCM3-affected individuals tended to show a higher median total number of CCM (12.0 vs 7.0, *P* = 0.147) as well as a higher median number of CCM in the posterior fossa (2.4 vs 1.5, *P* = 0.980) and brainstem (1.0 vs 0.7, *P* = 0.800) in their first available brain MRI when compared with subjects with other known genotypes, although not statistically significant.

**Table 2:** Imaging features of CCM detected at first brain MRI and with symptomatic hemorrhage identified at diagnosis and follow-up

CCM identified at first brain MRI *n* = 587	
Location, *n* (%)	
Cerebral lobes	492 (83.8)
Nucleocapsular/Thalamic	23 (3.9)
Brainstem	27 (4.6)
Cerebellum	39 (6.6)
Intra-ventricular	6 (1.0)
Modified Zabramski type, *n* (%)	
I	20 (3.4)
II	116 (19.8)
III	79 (13.5)
IV	362 (61.7)
V	10 (1.7)
Size in mm (except type IV CCM), median (IQR)	7.0 (1–52)
Giant lesions	5 (0.9)
CASH identified at diagnosis and follow-up *n* = 23	
Location, *n* (%)	
Cerebral lobes	14 (61.0)
Nucleocapsular/thalamic	1 (4.4)
Brainstem	5 (21.7)
Cerebellum	2 (8.7)
Spinal cord	1 (4.4)
Modified Zabramski type, *n* (%)	
I	6 (26.1)
V	17 (73.9)
Size in mm, median (IQR; range)	27 (25;8–52)
Multilocular morphology, *n* (%)	12 (52.2)
Hemossiderin ring, *n* (%)	15 (65.2)
Fluid–fluid levels, *n* (%)	13 (56.5)
T1 hyperintense perilesional sign, *n* (%)	15 (65.2)
Perilesional edema, *n* (%)	16 (69.6)
Associated DVA	0 (0.0)

Legend: *CASH*, cavernous angioma with symptomatic hemorrhage; *DVA*, developmental venous anomaly; *FU*, follow-up; *IQR*, interquartile range

Longitudinal brain MRs were available in 38 (92.7%) subjects, either performed as a scheduled examination or in the emergency setting due to new neurological events, with a median follow-up brain MRI time of 49.6 months (IQR = 68.6; range: 3.6–129.1). Fifteen (39.5%) developed at least 1 de novo CCM (median lesions per patient = 4.5; range: 1–16), for a total of 86 new lesions identified in 180.36 person-years of brain MRI follow-up, yielding an annual new lesion rate of 47.7%.

### Spine MR and CM

Twenty-three (56.1%) of the subjects had at least one available whole-spine MR for review (total = 44 studies performed). The mean age at the first available spine MR was 8.0 years (IQR = 5.0; range: 0.8–16.8 years). Retrospective longitudinal spine MR was additionally available in 11/23 (47.8%) of the cases (range: 2–7), with a median follow-up spine MR time of 39.5 months (range: 3–123.8). A total of *n* = 4 spinal CM were detected in three different patients (13%), one of them appearing de novo. Except for the single case presenting with a spine-related CASH that was previously described, the remaining spinal CM were asymptomatic.

### CASH lesions

**Fig. 1:**
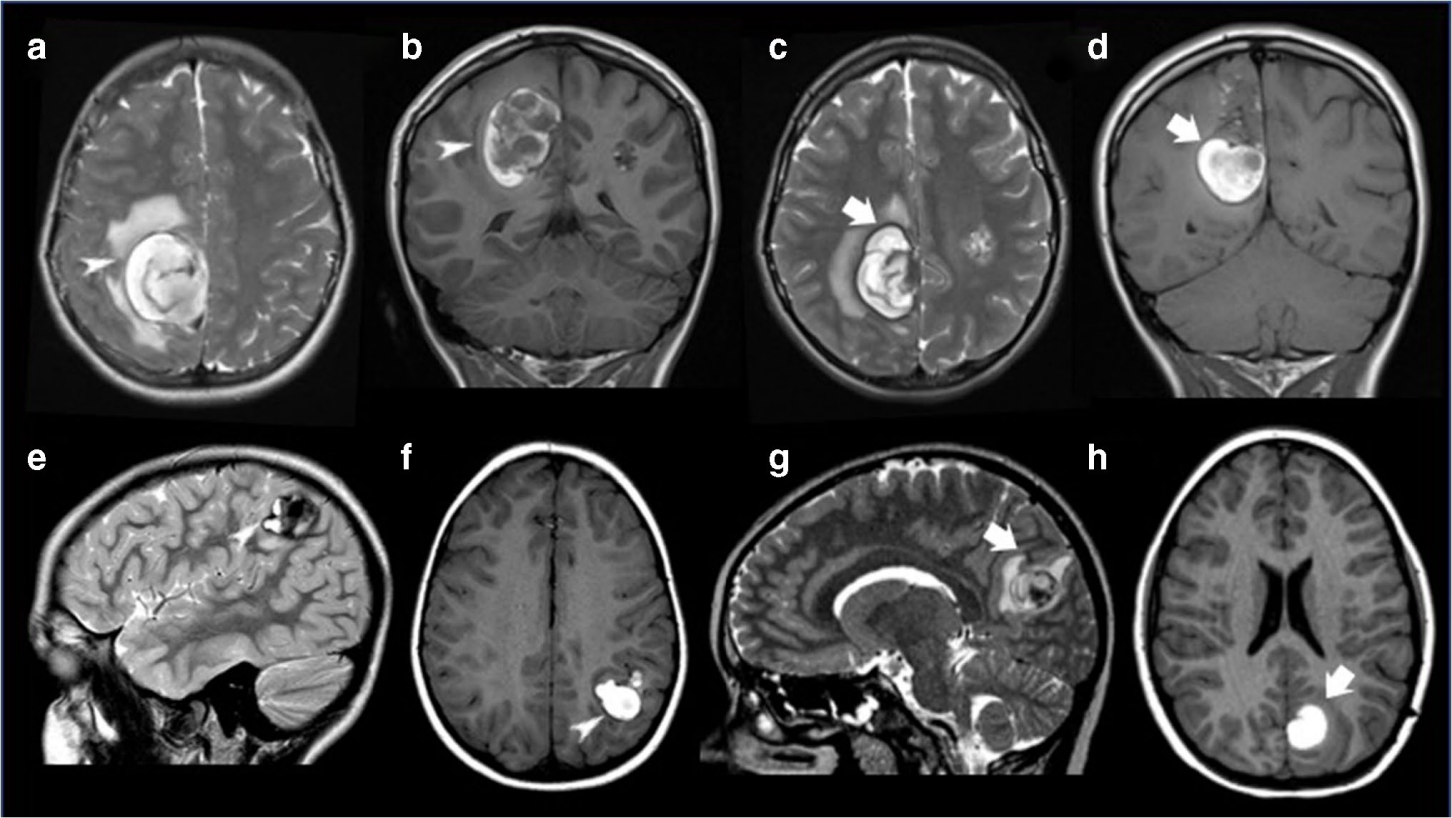
Examples of symptomatic hemorrhagic brain cavernous malformations. Brain MR (**a**, **b**) performed in the emergency setting in a 15-year-old girl with familial cerebral cavernous malformation syndrome due to a proven CCM1 mutation including axial T2 TSE (**a**) and coronal T1 SE (**b**) demonstrates an acute right parietal symptomatic hemorrhagic cerebral cavernous malformation (white arrowheads) with multiloculate appearance and complete hemosiderin ring as well as surrounding edema. No surgical treatment was performed. Follow-up brain MR of the same patient (**c**, **d**) performed after 22 months due to development of new acute neurological symptoms, including axial T2 TSE (**c**) and coronal T1SE (**d**), reveals symptomatic re-hemorrhage of the same cavernous malformation (white arrows), that was subsequently resected. Brain MR (**e**, **f**) performed in the emergency setting in a 7-year-old boy with familial cerebral cavernous malformation syndrome due to a proven CCM1 mutation including sagittal T2 TSE (e) and axial T1 SE (**f**) demonstrates an acute left parietal symptomatic hemorrhagic cerebral cavernous malformation (white arrowheads) with multiloculate appearance, complete hemosiderin ring, fluid–fluid levels and a small component of surrounding edema. This lesion was surgically removed with complete resection. Follow-up brain MR (**g**, **h**) performed after 50 months due to development of new acute neurological symptoms, including change to sagittal T2 TSE (**g**) and axial T1SE (**h**), shows a de novo cavernous malformation with signs of acute hemorrhage (white arrows), that was also subsequently resected

Imaging details of CASH lesions (*n* = 23), identified either at clinical presentation (*n* = 15) or during follow-up (*n* = 8), are also presented in Table 2 and some examples are illustrated in Fig. 1. Briefly, CASH were more commonly cerebral (*n* = 14, 61.0%), multilocular (*n* = 12, 52.2%), and had fluid–fluid levels (*n* = 13, 56.5%), hemosiderin ring (*n* = 15, 65.2%), perilesional edema (*n* = 17, 73.9%), and/or the T1-hyperintensity sign (*n* = 15, 65.2%). In no case, an associated DVA was found. Out of the eight CASH identified during retrospective longitudinal evaluation, 3 were caused by re-hemorrhage of a previous CASH, another 3 corresponded to a first symptomatic hemorrhagic event in known CCM, and 2 were in de novo CCM.

### *Annual prospectivesymptomatic cerebral hemorrhage rates*

In the 5-year follow-up, seven symptomatic hemorrhagic events occurred during 140.5 person-years, corresponding to a 5-year annual prospective symptomatic hemorrhagic rate of 5.0% (95% CI: 2.4–10.5). Other stratified hemorrhage risks per year per patient within this period are presented in Table 3.

**Table 3:** Hemorrhage risk per year per patient during 5-year follow-up

Variable	Total person-years	No. of symptomatic hemorrhagic events	Incidence rate (95% CI)
Gender			
Male	82.7	4	4.8 (1.8–14.5)
Female	57.9	3	5.2 (1.7–16.1)
Ethnic origin			
Caucasian	108.9	6	5.5 (2.5–12.3)
Non-Caucasian	31.6	1	3.2 (0.5–22.5)
Age at presentation			
< 6 years	53.1	2	3.8 (1.0–15.1)
$\ge$ 7 years	87.4	5	5.7 (2.4–13.7)
Positive family history			
Yes	93.1	6	6.4 (2.9–15.8)
No	47.4	1	2.1 (0.3–14.3)
Genotype			
CCM1	53.1	3	5.7 (1.8–17.5)
CCM2	16.3	2	12.3 (3.1–49.0)
CCM3	25.8	1	3.9 (0.6–27.5)
Presentation mode			
Symptomatic hemorrhage	49.2	5	10.2 (4.2–24.4)
Other types of presentation	91.3	2	2.2 (0.6–8.8)
$\ge$ 1 extra-CNS CM			
Yes	9.2	1	10.9 (1.5–77.4)
No	131.4	6	5.0 (2.4–10.5)
$\ge$ 10 CCM in the first brain MRI			
Yes	73.99	3	4.06 (1.31–12.57)
No	66.53	4	6.01 (2.26–16.02)
$\ge$ 1 CCM in the brainstem			
Yes	44.36	4	9.02 (3.39–24.03)
No	96.17	3	3.12 (1.01–9.6)
$\ge$ 1 CCM in the posterior fossa in the first brain MRI			
Yes	86.33	6	6.95 (3.12–15.47)
No	54.19	1	1.8 (0.26–13.10)
$\ge$ 1 Zabramski type II CCM in the first brain MRI			
Yes	105.95	5	5.79 (1.45–23.13)
No	34.57	2	4.72 (1.96–11.34)

Legend: *CM*, cavernous malformation; *CCM*, cerebral cavernous malformation; *CNS*, central nervous system; *CI*, confidence interval

In the maximum available follow-up time, one additional symptomatic event occurred, leading to a total of eight of such events occurring during 200.2 person-years and yielding an overall annual symptomatic hemorrhage rate of 4.0% (95% CI: 2.0–8.0) per person-year. The median time until the first prospective symptomatic hemorrhagic event during extended follow-up was 18.5 months (IQR = 31.3; range: 0.03–97.9).

### Cumulative risks of prospective hemorrhage

The cumulative risk of symptomatic hemorrhage was 7.3% (1.5–20.0) at 1 year, 14.6% (5.6–29.2) at 2 years, and 17.1% (7.1–31.2) at 5 years. The 5-year cumulative risk was higher in subjects with symptomatic hemorrhage at presentation (33.3%), *CCM2* genotype (33.3%), presence of at least one CCM in the brainstem at first brain MRI (26.7%), and positive family history (20.7%). Other stratified cumulative risks of hemorrhage at 5 years of follow-up are summarized in Table 4.

**Table 4:** Cumulative risk of symptomatic hemorrhage at 5 year follow-up of the 41 subjects stratified by clinical and genetic characteristics

Variable	*n*	No. of symptomatic hemorrhagic events during 5-year follow-up	5-year risk (95% CI)
Gender			
Male	22	4	18.2 (5.2–40.3)
Female	19	3	15.8 (3.4–39.6)
Ethnic origin			
Caucasian	30	6	20.0 (7.7–38.6)
Other	11	1	10.0 (2.3–41.3)
Age at presentation			
< 6 years	27	2	14.3 (1.8–42.8)
$\ge$ 7 years	14	5	18.5 (7.8–39.7)
Positive family history			
Yes	29	6	20.7 (8.0–39.7)
No	12	1	8.3 (2.1–38.5)
Genotype			
CCM1	17	3	17.6 (3.8–43.4)
CCM2	6	2	33.3 (4.3–77.7)
CCM3	6	1	14.3 (0.4–64.1)
Presentation mode			
Symptomatic hemorrhage	15	5	33.3 (11.8–61.7)
No hemorrhage	26	2	7.7 (1.0–25.1)
$\ge$ 1 extra-CNS CM			
Yes	2	1	50.0 (1.3–98.7)
No	39	6	15.4 (5.9–30.5)
$\ge$ 10 CCM at first brain MRI			
Yes	21	3	14.29 (3.05– 36.34)
No	20	4	20.00 (5.73–43.66)
$\ge$ 1 CCM in the brainstem at first brain MRI			
Yes	15	4	26.66 (7.79–55.1)
No	26	3	11.54 (2.45–30.15)
$\ge$ 1 CCM in the posterior fossa at first brain MRI			
Yes	26	6	23.08 (8.97–43.65)
No	15	1	6.67 (1.68–31.95)
$\ge$ 1 Zabramski type II CCM at first brain MRI			
Yes	31	5	16.13 (5.45–33.73)
No	10	2	20.0 (2.52–55.61)

Legend: *CM*, cavernous malformation; *CCM*, cerebral cavernous malformation; *CNS*, central nervous system; *CI*, confidence interval

**Fig. 2:**
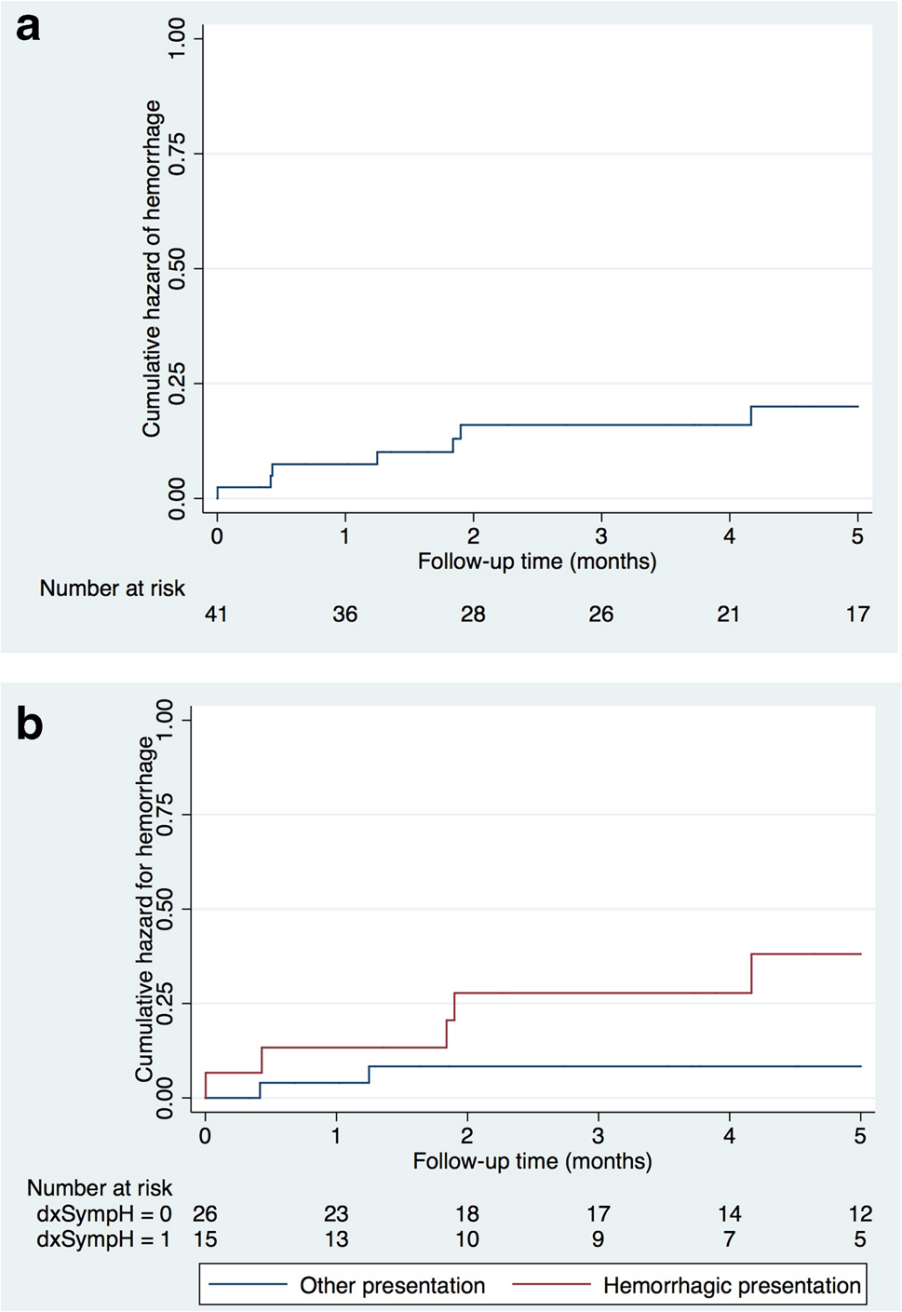
Cumulative hazard curves of hemorrhage of 41 children during 5-year follow-up for the entire cohort (**A**) and stratified by initial presentation of hemorrhage or other type of presentation (**B**)

A tendency towards a higher cumulative risk of symptomatic hemorrhage at 5 years of follow-up was observed in children presenting with prior symptomatic hemorrhage, with a borderline statistical significance (*P* = 0.0559) (Fig. 2).

### Predictors of symptomatic hemorrhage at presentation

In both uni- and multivariable analysis, none of the studied variables resulted in a significant independent predictor of symptomatic hemorrhagic presentation (Supplemental Table 2).

### Predictors of symptomatic hemorrhage at follow-up

In univariable analysis, the total number of CCM located in the brainstem (hazard ratio [HR] = 1.63, *P* = 0.003) and the total number of CCM located in the posterior fossa (HR = 1.37, *P* = 0.005) at the first brain MRI were significant risk factors of subsequent symptomatic hemorrhage at 5 years of follow-up. Multivariable analysis after adjusting for age at presentation and sex confirmed these variables as independent predictors of future symptomatic hemorrhage during that period (adjusted HR = 1.64 and 1.39 with *P* = 0.004 and 0.005, respectively). The symptomatic hemorrhagic presentation also showed a trend towards an increased risk for subsequent symptomatic hemorrhagic events when compared to other forms of presentation in both univariable and multivariable analysis, although without reaching statistical significance (adjusted HR = 4.33 and HR = 4.35, *P* = 0.008 and 0.08, respectively) (Table 5).

**Table 5:** Demographic and radiological risk factors of subsequent symptomatic hemorrhage at 5-year follow-up after initial diagnosis

	Univariable analysis		Multivariable analysis^a^	
Variable	Unadjusted HR (95% CI)	*P* value	Adjusted HR (95% CI)	*P* value
Male gender	1.00 (0.22–4.49)	1.00	0.99 (0.22–4.47)	0.99
Age at presentation, in years	1.02 (0.88–1.10)	0.82	1.02 (0.88–1.18)	0.82
Symptomatic hemorrhagic presentation	4.33 (0.84–22.3)	0.08	4.35 (0.84–22.50)	0.08
Positive family history	2.87 (0.35–23.88)	0.28	2.93 (0.35–24.50)	0.32
Genotype				
CCM1	Reference			
CCM2	2.25 (0.38–3.93)	0.38	2.97 (0.42–20.87)	0.27
CCM3	0.734 (0.08–7.07)	0.79	0.67 (0.07–6.71)	0.73
Presence of extra-CNS CM	2.51 (0.30–20.99)	0.40	2.70 (0.25–29.68)	0.84
Caucasian ethnicity	1.92 (0.23–15.00)	0.55	1.93 (0.23–16.28)	0.54
No. of CCM at first brain MRI	1.02 (0.98–1.06)	0.29	1.02 (0.98–1.06)	0.26
$\ge$ 10 CCM at first brain MRI	0.489 (1.12–1.78)	0.28	0.98 (0.35–2.77)	0.97
No. of CCM in the post fossa at first brain MRI	1.37 (1.10–1.71)	0.005*	1.39 (1.10–1.76)	0.005*
$\ge$ 1 CCM in the posterior fossa at first brain MRI	3.59 (0.33–4.74)	0.74	4.05 (0.45–36.37)	0.21
No. of brainstem CCM at first brain MRI	1.63 (1.18–2.26)	0.003*	1.64 (1.18–2.31)	0.004*
$\ge$ 1 CCM in the brainstem at first brain MRI	2.67 (0.60–11.97)	1.00	2.77 (5.91–12.98)	0.20
No. of Zabramski type II CCM at first brain MRI	1.06 (0.99–1.14)	0.12	1.07 (0.98–1.16)	0.12
$\ge$ 1 Zabramski type II CCM at first brain MRI	0.81 (0.16–4.18)	0.80	0.76 (0.14–4.16)	0.75
$\ge$ 1 de novo CCM at follow-up brain MRI	1.00 (0.22–4.48)	0.99	1.02 (0.87.1.19)	0.82
No. of de novo CCM at follow-up brain MRI	0.95 (0.76–1.19)	0.67	0.95 (0.75–1.20)	0.66

**Legend:** *CM*, cavernous malformation; *CCM*, cerebral cavernous malformation; *CI*, confidence interval; *CNS*, central nervous system; *FU*, follow-up; *HR*, hazard ratio; *IQR*, interquartile range

^a^Adjusted for age at presentation and gender

^*^Statistically significant value

## Discussion

In this multicenter study evaluating the natural history of pediatric FCCM in 41 children with a confirmed diagnosis of FCCM and incorporating standard definitions of symptomatic hemorrhage, we found that the 1-, 2-, and 5-year cumulative risks of hemorrhage were 7.3%, 14.6%, and 17.1%, respectively.

It has been previously described for the general population that the probability of symptomatic hemorrhage in CCM increases over time in the general population, especially during the first 2 years after hemorrhagic presentation, a phenomenon known as “temporal clustering” [8, 11, 12, 31]. This finding was also demonstrated in a pediatric cohort study involving children with different CCM subtypes [23] and confirmed in our cohort, including a homogeneous sample of children with FCCM. Indeed, our results show that although the cumulative risks of symptomatic hemorrhage increase over time in affected patients, they grow much faster in the first 2 years after the presentation of the disease (14.6% at 2 years vs 17.1% at 5 years after presentation). The transversality of temporal clustering suggests that it occurs irrespectively of age at presentation or CCM subtype.

We have also demonstrated that the annual symptomatic hemorrhage rate for our entire cohort at 5-year follow-up after diagnosis was 5.0% per person-year. When compared to the cumulative risk value previously presented, the annual symptomatic hemorrhage rate is a more robust measure, as it takes into account losses to follow-up and when events occur, although its interpretation is less intuitive. The influence of age at presentation and familial subtype in the natural history of subjects with one or multiple CCM remains unclear. Indeed, some authors have reported an increased symptomatic bleeding rate in children with CCM when compared to adults [19], while others have not [20, 23]. Moreover, the familial disease has also been linked to an increased symptomatic hemorrhagic risk [16] in the majority of, but not all [21], published studies. These divergent results are justified, at least in part, by methodological differences between the studies, including distinct sample sizes and eligibility criteria, variable proportions of CCM subtypes (familial vs sporadic), modes of presentation (hemorrhagic vs non-hemorrhagic), inconsistent methods of risk calculation (lifetime vs prospective approaches), and heterogenous definitions of hemorrhage [21, 27]. Our results support similar 5-year annual and cumulative symptomatic hemorrhagic risks in pediatric FCCM subjects treated surgically based on clinical judgement in tertiary centers, compared to children and adults with all types of CCM. These findings are in line with the most recent studies on this topic [23, 32]. However, the comparison of results between adult and pediatric cohorts should be made with caution. Indeed, mild symptomatic CCM-related neurologic events may be more commonly missed at young ages, especially if neurological manifestations were transient, insidious, or non-specific, as more frequently occurs in this age group [33]. Additionally, the threshold for a given CCM to become clinically symptomatic in pediatric patients may be higher than for adults, due to pediatric immaturity and/or increased cerebral plasticity of children [34]. This theory can be supported by the fact that the median size of CCM at presentation in children tends to be larger than in adults [28]. In addition, giant CCM are also more commonly found in children [28, 35] and both findings are corroborated by our results. Although giant CCM have primarily been described in sporadic patients, they can also occur in FCCM, as seen in five of our cases and previously reported by Ozgen et al. [28].

Another factor that may have influenced our results is the overall high rate of surgical treatment in our cohort (46%) when compared with other familial and/or pediatric CCM studies (up to 37.2%) [19, 20, 23, 32]. There are currently no standard treatment guidelines specific for pediatric FCCM and indications for surgical treatment of accessible lesions remain personalized and dependent upon multiple factors, including the individual surgeon’s practice standards, patient’s insurance, family preference, presentation mode and type, and severity of clinical manifestations at follow-up. The more aggressive approach followed in our referral tertiary pediatric hospitals likely reflects the high surgical training, volume, and expertise available in our centers, and likely contributed to fewer prospective symptomatic hemorrhagic events during follow-up. Planned surgical removal of CCM suspected of high-hemorrhagic risk (namely lesions with dynamic asymptomatic progressive changes detected on routine follow-up imaging or large size) was performed in some of our cases and likely positively modified the natural history of this disease.

The percentage of children in our cohort presenting with symptomatic hemorrhage is lower than reported in most previously published series including subjects with variable ages [15, 16, 20, 22, 23, 32]. This is probably due to the high rate of known positive family history of CCM in our cohort, leading to a higher index of suspicion in cases of mild or non-specific complaints, such as headaches, and/or screening brain MRIs in asymptomatic subjects. As prior symptomatic hemorrhage has been described by most authors as an independent risk factor for future symptomatic hemorrhagic events [8, 11, 12, 22, 23], the intrinsic baseline characteristics of our subjects may have led to our underestimation of the prospective hemorrhagic rates/cumulative risks. We also found that symptomatic hemorrhage at presentation rises the annual hemorrhagic rate and the 5-year cumulative risk of symptomatic hemorrhagic events of affected subjects when compared with other forms of presentation, although not reaching statistical significance as a predictor variable, most likely due to the sample size.

In our study, children with pathogenic loss-of-function variants in the *CCM2* gene also demonstrated higher annual hemorrhagic rates and 5-year cumulative risk of symptomatic hemorrhage when compared with subjects with *CCM1* or *CCM3* genetic variants. This association lacked statistical significance and therefore we cannot exclude that these results are due to chance, especially in the setting of the small number of subjects in our cohort harboring CCM2 genetic variants. All three *CCM1-3* genes encode components of a heterotrimeric CCM protein complex involved in endothelial stabilization but each of them has additional cellular functions. More specifically, CCM2 encodes malcavernin that acts as bridge allowing interaction between the other two CCM proteins and also influences β-catenin and Wnt signaling, causing MEKK3 inhibition and promotion of RhoA degradation [4]. Although few studies have specifically focused on *CCM2-*related disease, this genetic form has been so far considered a milder form of FCCM [36]. Instead, previous case series have described a more aggressive clinical course in patients who were carriers of *CCM3* variants [36–39] when compared with patients with other FCCM genotypes, although this feature has not been confirmed by all authors [40]. In our cohort, although the total number of CCM, posterior fossa CCM, and brainstem CCM was indeed higher in patients with CCM3 variants, differences did not reach statistical significance. In addition, the proportion of our subjects with symptomatic hemorrhagic presentation and moderate to severe neurological deficit at last follow-up or death was lower in CCM3 than in other FCCM disease-causing genes; however, also without statistical significance. To the best of our knowledge, no natural history study of FCCM comparing different genotypes has been previously published. Notably, 5/6 cases with variants of CCM2 had no single nucleotide variants but deletions of variable size that can also encompass additional flanking genes, including *GLI3*, leading to two cases of Greig cephalopolysyndactyly syndrome [30, 41]. This might have contributed to the disease phenotype and eventually influenced the longitudinal evolution of CCM in our cohort. The role of each gene in the natural history and long-term outcome of subjects with FCCM should be therefore further investigated in larger samples.

Our results also show that the first brain MRI can provide important information that can aid in the prognostication of future symptomatic hemorrhagic events in children with pediatric FCCM. Indeed, both the number of brainstem CCM and posterior fossa CCM at first brain MRI were significant independent predictors of prospective symptomatic hemorrhage events in our study. In contrast, the total number of CCM and the total number of Zabramsky type II lesions (either as continuous or dichotomized variables) played no significant role. These findings are in line with previous studies (including a meta-analysis) indicating that brainstem CCM location has an increased risk of symptomatic hemorrhage [8, 11, 12, 21]. However, it remains unclear whether brainstem CCM are intrinsically more prone to bleed or if there is an overestimation of the real hemorrhagic rate of CCM in this eloquent region [21]. Aiming to clarify this question, we believe that current reporting standards of hemorrhage from CCM [18] should be reviewed, recommending individualized reporting of both symptomatic and asymptomatic hemorrhage rates per patient and per lesion in future studies.

Differently from previous reports [23, 42], we could not find significant independent predictors of symptomatic hemorrhagic presentation in our cohort, most likely due to the sample size.

As expected in FCCM, the vast majority of our subjects had multiple CCM lesions at first brain MRI, even when the exam was performed during early infancy. A recent report has shown that CCM may even be detected in utero in familial cases using fetal MRI [43]. However, it is important to bear in mind that even isolated CCM lesions in children may be familial, as seen in one of our cases. In line with previous studies [16, 17, 27, 44], we have also detected the imaging appearance of new CCM over time (reaching an 8.3% annual rate per patient-year) and some of these de novo CCM were responsible for symptomatic hemorrhagic events. This dynamic activity is consistently higher in familial cases when compared to sporadic ones [16, 17, 27, 44]. Nevertheless, direct comparison between studies concerning the rate of development of new lesions is hampered by methodological differences, as reviewed in a recent meta-analysis by Taslimi et al. [27].

CASH lesions in our cohort were overall more commonly located supratentorially (probably due to the high supratentorial/infratentorial ratio of lesion counts) and their median size was larger than the median size of all CCM (excluding type IV lesions). These usually demonstrated a hemosiderin ring and extracapsular hemorrhagic extension with associated vasogenic edema as well as imaging features commonly described in large-size/giant CCM, namely multilobulated morphology and internal fluid levels [28]. In addition, the majority but not all CASH exhibited the T1 hyperintense perilesional signal, an imaging feature with moderate sensitivity and high sensitivity for the diagnosis of hemorrhagic CCM [29]. Nevertheless, it is important to be aware that this imaging sign may also be evident in other lesions, such as melanoma and other hemorrhagic metastasis [45].

Based on the results of this study as well as other recently published papers focusing on FCCM in children, adults, and/or mixed populations, we suggest that patients with suspected and/or confirmed FCCM should be imaged at the best available MR scanner (ideally in a 3.0 Tesla unit) with standard imaging technique [9, 46–48]. The brain MR protocol should include a T1 3D sequence, axial and coronal T2WI, axial or 3D FLAIR, and at least one GRE sequence, preferably SWI [46, 47] Whole-spine MR should be additionally performed at diagnosis regardless of the presentation age as a screening modality, including a T1 TSE, a T2 TSE, and at least one GRE sequence, ideally a 3D T2 Multi-Echo Data Image Combination (MEDIC) [9, 48] Serial spine MR imaging with a similar protocol should be also considered even in patients with initially negative spine MR studies.

Our study has some limitations, including its retrospective design and tertiary center referral bias. In addition, our cohort was also predominantly composed of Caucasian subjects, which may limit generalizability to subjects with other ethnic backgrounds, including the commonly reported Hispanic FCCM population. As guidelines for brain imaging in pediatric FCCM are currently not well defined [2], subjects were imaged over time at inconsistent time points and without standard MR techniques (including variations in the MR scanner magnetic field strength and/or the protocol). This may have limited the imaging assessment, since the identification of CCM is strongly dependent on technical parameters. Moreover, due to the retrospective nature of the study, we have not evaluated advanced imaging techniques such as perfusion and permeability MR that have been recently described as biomarkers of CCM activity and predictors of lesional growth and hemorrhage [49–51]. Another possible limitation of our results includes the 39% rate of loss to follow-up at 5 years that occurred mainly due to adult care transferal. Finally, the number of bleeding events was rather low during follow-up, limiting the number of possible variables and increasing the 95% CI in the Cox proportion regression analysis model. Nevertheless, to the best of our knowledge, this is a natural history study conducted in the largest pediatric FCCM cohort reported to date, including a comprehensive clinical, genetic, and imaging evaluation. More specifically, when compared with the recent paper by Santos et al. [23], our study includes a larger number of patients with pediatric FCCM (41 vs 35) and provides a better clinical characterization of the cohort as well detailed neuroimaging assessment of their CCM, with special emphasis on de novo CCM and symptomatic hemorrhagic lesions. In addition, and contrarily to the paper by Santos et al. [23], we specify the pathogenic variants of all subjects with genetic FCCM confirmation and evaluate the relationship between the genetic subtypes of FCCM and the prospective symptomatic hemorrhagic events.

## Conclusions

Pediatric FCCM seem to have an overall similar 5-year annual and cumulative symptomatic hemorrhagic risks in subjects treated according to clinical judgement (including medical and/or surgical approach) compared to children with sporadic CCM and adults with either familial or sporadic disease. In addition, children with FCCM can be stratified to predict longitudinal symptomatic hemorrhage risks according to baseline clinical and imaging features, allowing a differentiated treatment strategy. Future prospective multicenter studies in pediatric FCCM using standardized MR performed at fixed time points and including advanced neuroimaging techniques and genetic investigation would be advisable to increase our current knowledge and test new predictors of both symptomatic and asymptomatic hemorrhagic events as well as the overall neurological outcome.

## Supplementary Information

Below is the link to the electronic supplementary material.

Supplementary file1 (DOCX 20.5 KB)

## Abbreviations

CASH: Cavernous angioma with symptomatic hemorrhage
CCM: Cerebral cavernous malformation
CM: Cavernous malformation
DVA: Developmental venous anomaly
FCCM: Familial cerebral cavernous malformation
SWI: Susceptibility-weighted image

## References

[1] Zafar A, Quadri SA, Farooqui M (2019). Familial cerebral cavernous malformations. Stroke.

[2] Akers A, Al-Shahi Salman R, Awad IA (2017). Synopsis of guidelines for the clinical management of cerebral cavernous malformations: consensus recommendations based on systematic literature review by the angioma alliance scientific advisory board clinical experts panel. Neurosurgery.

[3] Boulouis G, Blauwblomme T, Hak JF (2019). Nontraumatic pediatric intracerebral hemorrhage. Stroke.

[4] Riolo G, Ricci C, Battistini S (2021). Molecular genetic features of cerebral cavernous malformations (CCM) patients: an overall view from genes to endothelial cells. Cells.

[5] Ren AA, Snellings DA, Su YS (2021). PIK3CA and CCM mutations fuel cavernomas through a cancer-like mechanism. Nature.

[6] Hong T, Xiao X, Ren J (2021). Somatic MAP3K3 and PIK3CA mutations in sporadic cerebral and spinal cord cavernous malformations. Brain.

[7] Peyre M, Miyagishima D, Bielle F (2021). Somatic PIK3CA mutations in sporadic cerebral cavernous malformations. N Engl J Med.

[8] Taslimi S, Modabbernia A, Amin-Hanjani S (2016). Natural history of cavernous malformation. Neurology.

[9] Mabray MC, Starcevich J, Hallstrom J (2020). High prevalence of spinal cord cavernous malformations in the familial cerebral cavernous malformations type 1 cohort. AJNR Am J Neuroradiol.

[10] Manole AK, Forrester VJ, Zlotoff BJ (2020). Cutaneous findings of familial cerebral cavernous malformation syndrome due to the common Hispanic mutation. Am J Med Genet A.

[11] Al-Shahi Salman R, Hall JM, Horne MA (2012). Untreated clinical course of cerebral cavernous malformations: a prospective, population-based cohort study. Lancet Neurol.

[12] Horne MA, Flemming KD, Su IC (2016). Clinical course of untreated cerebral cavernous malformations: a meta-analysis of individual patient data. Lancet Neurol.

[13] Zabramski JM, Wascher TM, Spetzler RF (1994). The natural history of familial cavernous malformations: results of an ongoing study. J Neurosurg.

[14] Labauge P, Laberge S, Brunereau L (1998). Hereditary cerebral cavernous angiomas: clinical and genetic features in 57 French families. Lancet.

[15] Brunereau L, Labauge P, Tournier-Lasserve E (2000). Familial form of intracranial cavernous angioma: MR imaging findings in 51 families. Radiology.

[16] Labauge P, Brunereau L, Lévy C (2000). The natural history of familial cerebral cavernomas: a retrospective MRI study of 40 patients. Neuroradiology.

[17] Brunereau L, Levy C, Laberge S (2000). De novo lesions in familial form of cerebral cavernous malformations: clinical and MR features in 29 non-Hispanic families. Surg Neurol.

[18] Al-Shahi Salman R, Berg MJ, Morrison L (2008). Hemorrhage from cavernous malformations of the brain: definition and reporting standards. Stroke.

[19] Acciarri N, Galassi E, Giulioni M (2009). Cavernous malformations of the central nervous system in the pediatric age group. Pediatr Neurosurg.

[20] Al-Holou WN, O’Lynnger TM, Pandey AS et al (2012) Natural history and imaging prevalence of cavernous malformations in children and young adults. Clinical article. J Neurosurg Pediatr 9:198–205 10.3171/2011.11.PEDS11390 22295927

[21] Nikoubashman O, Di Rocco F, Davagnanam I (2015). Prospective hemorrhage rates of cerebral cavernous malformations in children and adolescents based on MRI appearance. AJNR Am J Neuroradiol.

[22] Gross BA, Du R, Orbach DB (2016). The natural history of cerebral cavernous malformations in children. J Neurosurg Pediatr.

[23] Santos AN, Rauschenbach L, Saban D (2022). Natural course of cerebral cavernous malformations in children: a five-year follow-up study. Stroke.

[24] Polster SP, Stadnik A, Akers AL (2019). Atorvastatin treatment of cavernous angiomas with symptomatic hemorrhage exploratory proof of concept (AT CASH EPOC) Trial. Neurosurgery.

[25] Shenkar R, Peiper A, Pardo H (2019). Rho kinase inhibition blunts lesion development and hemorrhage in murine models of aggressive Pdcd10/Ccm3 disease. Stroke.

[26] Lanfranconi S, Scola E, Bertani GA (2020). Propranolol for familial cerebral cavernous malformation (Treat_CCM): study protocol for a randomized controlled pilot trial. Trials.

[27] Taslimi S, Ku JC, Modabbernia A, Macdonald RL (2019). Hemorrhage, seizures, and dynamic changes of familial versus nonfamilial cavernous malformation: systematic review and meta-analysis. World Neurosurg.

[28] Ozgen B, Senocak E, Oguz KK (2011). Radiological features of childhood giant cavernous malformations. Neuroradiology.

[29] Yun TJ, Na DG, Kwon BJ (2008). A T1 hyperintense perilesional signal aids in the differentiation of a cavernous angioma from other hemorrhagic masses. AJNR Am J Neuroradiol.

[30] Bilguvar K, Bydon M, Bayrakli F (2007). A novel syndrome of cerebral cavernous malformation and Greig cephalopolysyndactyly: Laboratory investigation. J Neurosurg.

[31] Barker FG, Amin-Hanjani S, Butler WE (2001). Temporal clustering of hemorrhages from untreated cavernous malformations of the central nervous system. Neurosurgery.

[32] Santos AN, Rauschenbach L, Saban D (2022). Multiple cerebral cavernous malformations: clinical course of confirmed, assumed and non-familial disease. Eur J Neurol.

[33] Houwing ME, Grohssteiner RL, Dremmen M (2022). Silent cerebral infarcts in patients with sickle cell disease: a systematic review and meta-analysis. BMC Med.

[34] Bonfanti L, Charvet CJ (2021). Brain plasticity in humans and model systems: advances, challenges, and future directions. Int J Mol Sci.

[35] Wang C, Zhao M, Wang J (2018). Giant cavernous malformations: a single center experience and literature review. J Clin Neurosci.

[36] Denier C, Labauge P, Bergametti F (2006). Genotype-phenotype correlations in cerebral cavernous malformations patients. Ann Neurol.

[37] Riant F, Bergametti F, Fournier HD (2013). CCM3 mutations are associated with early-onset cerebral hemorrhage and multiple meningiomas. Mol Syndr.

[38] Shenkar R, Shi C, Rebeiz T (2015). Exceptional aggressiveness of cerebral cavernous malformation disease associated with PDCD10 mutations. Genet Med.

[39] Merello E, Pavanello M, Consales A (2016). Genetic screening of pediatric cavernous malformations. J Mol Neurosci.

[40] Cigoli MS, Avemaria F, De Benedetti S (2014). PDCD10 gene mutations in multiple cerebral cavernous malformations. PLoS One.

[41] Liquori CL, Berg MJ, Squitieri F (2007). Deletions in CCM2 are a common cause of cerebral cavernous malformations. Am J Hum Genet.

[42] Kashefiolasl S, Bruder M, Brawanski N (2018). A benchmark approach to hemorrhage risk management of cavernous malformations. Neurology.

[43] Cheng D, Shang X, Gao W (2021). Fetal familial cerebral cavernous malformation with a novel heterozygous KRIT1 variation. Neurology.

[44] Nikoubashman O, Wiesmann M, Tournier-Lasserve E (2013). Natural history of cerebral dot-like cavernomas. Clin Radiol.

[45] Nabavizadeh SA, Pechersky D, Schmitt JE (2017). Perilesional hyperintensity on T1-weighted images in intra-axial brain masses other than cavernous malformations. J Neuroimaging.

[46] de Souza JM, Domingues RC, Cruz LCHJ (2008). Susceptibility-weighted imaging for the evaluation of patients with familial cerebral cavernous malformations: a comparison with T2-weighted fast spin-echo and gradient-echo sequences. AJNR Am J Neuroradiol.

[47] Sparacia G, Speciale C, Banco A (2016). Accuracy of SWI sequences compared to T2∗-weighted gradient echo sequences in the detection of cerebral cavernous malformations in the familial form. Neuroradiol J.

[48] Geraldo AF, Luis A, Alves CAPF (2022). Spinal involvement in pediatric familial cavernous malformation syndrome. Neuroradiology.

[49] Mikati AG, Tan H, Shenkar R (2014). Dynamic permeability and quantitative susceptibility related imaging biomarkers in cerebral cavernous malformations. Stroke.

[50] Girard R, Fam MD, Zeineddine HA (2017). Vascular permeability and iron deposition biomarkers in longitudinal follow-up of cerebral cavernous malformations. J Neurosurg.

[51] Sone JY, Hobson N, Srinath A (2022). Perfusion and permeability MRI predicts future cavernous angioma hemorrhage and growth. J Magn Reson Imaging.

