# Supplementary material for: Natural history of familial cerebral cavernous malformation syndrome in children: a multicenter cohort study
Source: Neuroradiology. 2022 Oct 6;65(2):401–14. doi: 10.1007/s00234-022-03056-y (PMC9859903; doi:10.1007/s00234-022-03056-y)
Supplement: Supplementary file 1 — Supplementary file1 (DOCX 20.5 KB) [file 234_2022_3056_MOESM1_ESM.docx]

Supplementary Material

**Supplemental Table 1.** Comparison between subjects surgically and conservatively treated.

|  | **Surgically treated**  **subjects**  **N=19 (46.3%)** | **Conservatively treated subjects**  **N= 22 (53.7%)** | **P-value** |
| --- | --- | --- | --- |
| **Male, n (%)** | 8 (42.1) | 14 (63.6) | 0.167 |
| **Age at initial clinical presentation in years, median (IQR)** | 7.8 (4.5-12.8)  Range: 0.4-15.8 | 6.8 (3.0-12.3)  Range: 0.8-17.3 | 0.657 |
| **Genotype, n (%)**  C*CM1*  CCM2^a^  C*CM3*  *CCM1-3* testing negative/not performed/pending | 8 (42.1)  3 (15.8)  3 (15.8)  5 (26.3) | 9 (40.9)  3 (13.6)  4 (18.2)  6 (27.3) | 1.0 |
| **Positive family history, n (%)** | 24 (73.7) | 15 (68.2) | 0.699 |
| **Ethnic origin, n (%)**  Caucasian  Other | 15 (79.0)  4 (21.1) | 15 (68.2)  7 (31.8) | 0.499 |
| **Presentation mode, n (%)**  Symptomatic hemorrhage of the CNS^b^  Other type of clinical presentation | 10 (52.6)  9 (47.4) | 5 (22.7)  17 (77.3) | 0.047* |
| $\boldsymbol{\geq}$ **1 extra-CNS CM, n (%)** | 1 (5.3) | 1 (4.6) | 1.0 |
| **Neurological assessment at last clinical FU, n (%)**  Normal/mild  Moderate/severe impairment or death | 13 (68.4)  6 (31.6) | 19 (86.4)  3 (13.6) | 0.166 |
| **Seizures at last clinical FU, n (%)** | 8 (42.1) | 4 (18.2) | 0.167 |
| **Clinical FU time in months, median (IQR)** | 46.8 (22.3-97.9)  Range:8.9-143.4 | 58.9 (37.1-92.3)  Range: 4.0-205.4 | 0.548 |
| **Nº CCM at first brain MRI, median (IQR)** | 6 (2-12; 1-80) | 12 (8-23; 2-67) | 0.0204* |
| **Presence of >=1 CCM in the posterior fossa at first brain MRI, n (%)** | 10 (52.6) | 16 (72.7) | 0.183 |

**Legend**: CM, cavernous malformation; CCM, cerebral cavernous malformation; CNS, central nervous system; FU- follow-up; IQR, interquartile range

^a^ includes 4 cases with a 7p deletion; ^b^ 1 case due to a SCCM-related hemorrhage

* statistically significant value

***Genetic analysis***

**DNA extraction:** Genomic DNA was isolated from 1 ml of peripheral blood using QIAamp® DNA Blood Midi kit (Qiagen), according to manufacturer&#39;s instructions. DNA was quantified using a Qubit™ dsDNA BR Assay Kit on a Qubit 2.0 Fluorometer (Life Technologies). **NGS Assay Design:** A next generation sequencing (NGS) custom-designed panel was created using the Ion AmpliSeq™ Designer v6.13 algorithm provided by Thermo Fisher Scientific (Carlsbad, CA, USA) in order to target the entire coding sequence (CDS) and 10 bases of the adjacent intronic regions of KRIT1/CCM1 (NM_194456; 19 exons), CCM2 (NM_031443; 10 exons), and CCM3/PDCD10 (NM_007217; 10 exons). The primers pools were composed by 44 amplicons showing the following features: 100% of total coverage, 0% missed regions, an amplicons range of 125- 275 bp and an exon padding of 10 bp. **Libraries preparation:** Multiplex PCR were performed manually using 10 ng of genomic DNA with a premixed primer pool and Ion AmpliSeq HiFi master mix (Ion AmpliSeq Library Kit 2.0). The amplicons were treated with 2µl FuPa reagent to partially digest the primer sequences and phosphorylate the amplicons. Amplicons were ligated to adapters with the diluted barcodes of Ion Xpress Barcode Adapters kit (Thermo Fisher Scientific, Inc.). The libraries were purified using the Agencourt AMPure XP Reagent (Beckmann Coulter, CA, USA). The concentration of the final libraries was determined by fluorescent measurement on Qubit 2.0 instrument (Life Technologies). **Template preparation, chip loading and sequencing:** The libraries were diluted to ~100 pM. The clonal amplification of barcoded DNA libraries onto ion spheres was performed on an Ion Chef™ Instrument using Ion 520™ Kit-Chef, according to the manufacturer&#39;s instructions (Thermo Fisher Scientific, Inc.). Template-positive spheres from barcoded libraries were loaded onto Ion 510™ Chips following the manufacturer&#39;s protocol, and sequencing was run on the Ion Gene Studio S5 system (Thermo Fisher Scientific, Inc.). **Data analysis:** DNA sequence reads were analyzed using both the Ion Reporter Software v.5.6 (Thermo Fisher Scientific, Inc.) and the CLC Genomics Workbanch 6.5.1 software (Qiagen). High-quality Single Nucleotide Variants (SNV) and insertion/deletion variants were strictly defined as: i) FILTER=PASS, ii) QUAL ≥100, iii) depth coverage ≥20X, and iv) variant fraction ≥20%. Bioinformatics tools were used to predict the effect of missense variants: Polyphen-2 (http://genetics.bwh.harvard.edu/pph2/index.shtml), SIFT (<http://sift.bii.astar.edu.sg/www/SIFT_seq_submit2.html>), MutPred (http://mutpred.mutdb.org/) and Mutation Taster (http://www.mutationtaster.org/). Splicing variants were studied using Human Splicing Finder, vs. 3.0 (<http://www.umd.be/HSF3/HSF.html>). **Variants validations:** The variants called by both softwares and having a predicted functional effect were validated by Sanger sequencing using High-Fidelity Platinum Master Mix (Invitrogen) for PCR and BigDye Terminator v1.1 kit (Life Technologies). Multiplex Ligation-Dependent Probe Amplification (MLPA) Assay MLPA was performed on those patients who were negative for mutations in CCM1, CCM2 and CCM3 genes using two MLPA probemix, P130 and P131, according to the standard MLPA protocol (MRC-Holland, Amsterdam, The Netherlands). Coffalyser software was used for analysis of peak values obtained from capillary electrophoresis on ABI 3100 Genetic Analyzer (Applied Biosystems). Mean cut-off for normalized peak height ratio of patient to the control sample was less than 0.7 in case of exon deletions and more than 1.30 in case of exon duplications.

*Brain MR and CCM*

A total of 199 brain MRI studies were available for review (median number per patient=4.0; range:1-16), 51 (25.6%) performed on a 1.5T magnet and 148 (74.4%) on a 3.0T magnet. A T2* and/or SWI sequence was respectively available in 80 (40.2%) and 136 (68.3%) studies, while both sequences obtained in 27 (13.9%) examinations. Contrast material was injected in 39 (19.6%) occasions.

**Supplemental Table 2.** Clinical risk of CCM-related symptomatic hemorrhage as mode of presentation.

|  | **Univariable analysis** | | **Multivariable analysis** ^a^ | |
| --- | --- | --- | --- | --- |
| **Variable** | **﻿Unadjusted OR (95% CI)** | **﻿P value** | **Adjusted OR (95% CI)** | **P value** |
| **Male gender** | 1.0 (0.3-3.5) | 0.975 | 0.99 (0.27-3.54) | 0.982 |
| **Age at presentation in years** | 1.0 (0.9-1.1) | 0.130 | 0.99 (0.88-1.13) | 0.910 |
| **Positive family history** | 2.1 (0.5-9.5) | 0.275 | 2.12 (0.47-9.54) | 0.329 |
| **Genotype**  CCM1  CCM2  CCM3 | Reference  0.56 (0.08-3.93)  0.45 (0.068-3.00) | Reference  0.890  0.246 | Reference  0.56 (0.08-3.98)  0.43 (0.64-3.02) | Reference  0.566  0.403 |
| **Presence of extra-CNS CM** | 1.8 (0.1-30.8) | 0.690 | 1.97 (0.10-38.86) | 0.656 |
| **Caucasian ethnicity** | 0.0 (0.2-4.3) | 0.749 | 1.00 (0.23-3.77) | 0.998 |

**Legend:** CM, cavernous malformation; CCM, cerebral cavernous malformation; CI, confidence interval; CNS, central nervous system; FU- follow-up; IQR, interquartile range; OR, Odds ratio

^a^ adjusted for age at presentation and gender
